# Biosynthesis of rare hexoses using microorganisms and related enzymes

**DOI:** 10.3762/bjoc.9.281

**Published:** 2013-11-12

**Authors:** Zijie Li, Yahui Gao, Hideki Nakanishi, Xiaodong Gao, Li Cai

**Affiliations:** 1 The Key Laboratory of Carbohydrate Chemistry and Biotechnology, Ministry of Education, School of Biotechnology, Jiangnan University, Wuxi, 214122, China; 2 School of Food Science and Technology, Jiangnan University, Wuxi, 214122, China; 3 Division of Mathematics and Science, University of South Carolina Salkehatchie, Walterboro, South Carolina, 29488, USA

**Keywords:** biosynthesis, enzyme, hexose, microorganism, rare sugars

## Abstract

Rare sugars, referred to as monosaccharides and their derivatives that rarely exist in nature, can be applied in many areas ranging from foodstuffs to pharmaceutical and nutrition industry, or as starting materials for various natural products and drug candidates. Unfortunately, an important factor restricting the utilization of rare sugars is their limited availability, resulting from limited synthetic methods. Nowadays, microbial and enzymatic transformations have become a very powerful tool in this field. This article reviews the biosynthesis and enzymatic production of rare ketohexoses, aldohexoses and sugar alcohols (hexitols), including D-tagatose, D-psicose, D-sorbose, L-tagatose, L-fructose, 1-deoxy-L-fructose, D-allose, L-glucose, L-talose, D-gulose, L-galactose, L-fucose, allitol, D-talitol, and L-sorbitol. New systems and robust catalysts resulting from advancements in genomics and bioengineering are also discussed.

## Introduction

Rare sugars are referred to as monosaccharides and their derivatives that rarely exist in nature (http://isrs.kagawa-u.ac.jp/activities.html) [1]. Seeberger et al. did some interesting statistical research on bacterial and mammalian glycomes, with one emphasis placed on the abundance of monosaccharide units (most abundant monosaccharides are listed therein) [2–3]. Rare sugars are thus classified by their low natural abundance, compared to those common sugars such as D-glucose, D-galactose, *N*-acetylglucosamine, etc that exist in significant amounts. Nonetheless, rare sugars can be applied in many areas ranging from foodstuffs to pharmaceutical and nutrition industry [4]. In recent years, the interest in rare carbohydrates and the nucleosides (e.g. L-nucleoside analogues) derived from them has substantially increased in medicine because these molecules are potential candidates of anticancer and antiviral drugs [4–6]. Unfortunately, the limited availability of these compounds restricts their potential applications and the chemical synthesis does not satisfy the increasing demand. Izumori et al. created beautiful “Izumoring” schemes displaying all rare sugars in tree form to illustrate possible strategies for the production of these monosaccharides [7]. Recently, inspired by the “Izumoring” tree, microbial and enzymatic transformations have become a very powerful tool for the synthesis of rare sugars owing to the advancements in genomics and an increasing availability of new and robust biocatalysts. Microbial transformation has the advantages of using cheaper catalysts without purification and the avoidance of cofactor recycling in vitro, while enzymatic conversion is more controllable and scalable. In addition, immobilized enzymes offer advantages over free and soluble enzymes in many aspects: 1) immobilized enzymes can be used repeatedly and the enzymatic processes can be operated continuously and readily controlled; 2) products can be easily harvested and purified; 3) enzyme properties (such as activity and stability) could be potentially improved through immobilization. In this review, we mainly focus on the biosynthesis and enzymatic production of rare hexoses, including ketohexoses, aldohexoses, and sugar alcohols (hexitols). To improve the production of these rare sugars, strategies for enhancing the catalytic efficiency and/or substrate selectivity of related enzymes are also discussed.

## Review

### I. Biosynthesis of rare ketohexoses

The most common ketohexoses (ketone-containing hexoses), each of which represents a pair of enantiomers (D- and L-isomers), include tagatose, psicose, sorbose, and fructose. Among the eight sugars, D-tagatose, D-psicose, D-sorbose, L-tagatose and L-fructose are generally regarded as rare sugars due to their low abundance in nature. In addition, 1-deoxy-L-fructose is also a very rare but important monosaccharide [8]. The biosynthesis of these rare ketohexoses using microorganisms and related enzymes is summarized below.

#### D-Tagatose

**Scheme 1:**
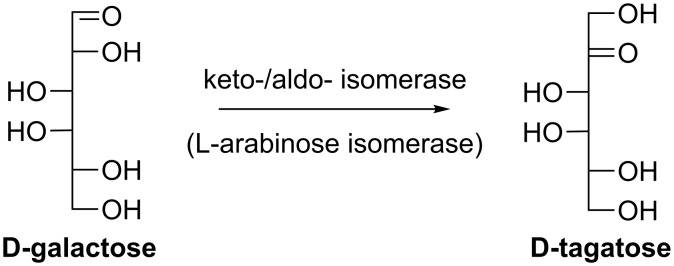
Synthesis of D-tagatose from D-galactose using L-arabinose isomerase.

D-Tagatose is a keto-/aldo-isomer of D-galactose. It has been approved by the FDA as a food additive and gained much attention owing to its properties and health benefits, including low calorie [9], no glycemic effect [10], promotion of weight loss [11], tooth care [12], and prebiotic [13]. D-Tagatose production through bioconversion has been extensively studied, particularly from D-galactose by L-arabinose isomerase (AI, EC 5.3.1.4) (Scheme 1) [14–20]. As the interconversion equilibrium between D-galactose and D-tagatose shifts toward D-tagatose at higher temperatures, biocatalysts with greater thermal stability are preferred. Thus AIs from thermophilic or hyperthermophilic bacteria are of special interest. It was reported that the highest level of D-tagatose production was 230 g/L from 500 g/L D-galactose catalyzed by an immobilized AI from *Geobacillus stearothermophilus* [21] and the highest yield was 68% at 80 °C by AI from *Thermotoga neapolitana* [22]. However, two main problems still exist for the AI-catalyzed D-tagatose synthesis. The catalytic efficiency of AI for D-galactose is relatively low compared to its natural substrate L-arabinose. Enzyme engineering has thus been reported to improve the efficiency of AI towards D-galactose [23]. Another challenge lies in the product purification due to very similar properties of D-galactose and D-tagatose. Liang et al. elegantly employed *Saccharomyces cerevisiae* to selectively consume D-galactose and thus D-tagatose could be obtained at above 95% purity [24].

#### D-Psicose

**Scheme 2:**
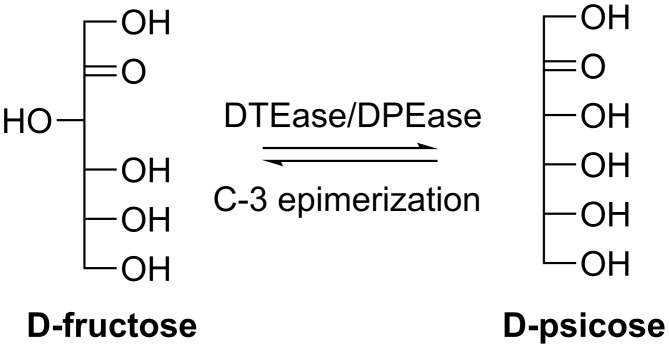
Synthesis of D-psicose from D-fructose using D-tagatose 3-epimerase/D-psicose 3-epimerase.

D-Psicose is a C-3 epimer of D-fructose and a potential sucrose substitute sweetening agent. The suppression of hepatic lipogenic enzymes activity of D-psicose has been noticed for its use as a non-caloric sweetener [25–26]. In addition, antioxidant properties have been observed for foods containing this rare sugar [25]. More interestingly, D-psicose has also been used to prepare several D-psicose containing disaccharides, aiming to learn the function of the rare sugar containing oligosaccharides and glycosides [27]. D-Psicose can be prepared through the epimerization of D-fructose at C-3 catalyzed by an ezyme of the D-tagatose 3-epimerase family (DTEase, EC 5.1.3.-), which is a commercially attractive enzymatic reaction for D-psicose production (Scheme 2). To date, five DTEases from different organisms have been characterized and employed for the D-psicose synthesis. Izumori et al. firstly characterized the DTEase from *Pseudomonas sp.* ST-24 [28] and utilized this enzyme in the mass production of D-psicose with a final concentration of 150 g/L [29]. They further packed the immobilized DTEase from *Pseudomonas sp.* ST-24 (on Chitopearl beads) into a column that could be continuously used for 10 days and 90 g of D-psicose were produced from 500 g of D-fructose as the starting material [30]. Kim et al. also characterized a putative DTEase from *Agrobacterium tumefaciens* [31] and due to its high substrate specificity towards D-psicose, this enzyme was renamed as D-psicose 3-epimerase (DPEase, EC 5.1.3.-).

It was discovered that borate could promote the conversion of D-fructose to D-psicose through the formation of a psicose–borate complex [32]. The maximum conversion yield of D-psicose in the presence of borate was about two-fold compared to the reaction without borate. Lim et al. established a stable immobilized *A. tumefaciens* DPEase system containing borate [33]. It could generate 441 g/L D-psicose from 700 g/L D-fructose for one batch, and the reaction performed in a packed-bed bioreactor could continuously produce 325 g/L D-psicose from 500 g/L D-fructose over a long time period. Zhang et al. characterized the third DTEase from *Rhodobacter sphaeroides* SK011 [34] and the specificity of this enzyme towards D-fructose was the highest under optimized conditions. Recently, Mu et al. characterized the fourth and fifth DTEase from *Clostridium cellulolyticum* H10 and *Clostridium scindens* 35704 respectively. As these two enzymes showed high preference towards D-psicose, they were both renamed as DPEase as well. The activity of these two enzymes is strictly metal-dependent and requires Mn^2+^ as the optimum cofactor. Under optimal conditions, the epimerization yields were 32% and 28%, respectively [35–36].

**Figure 1:**
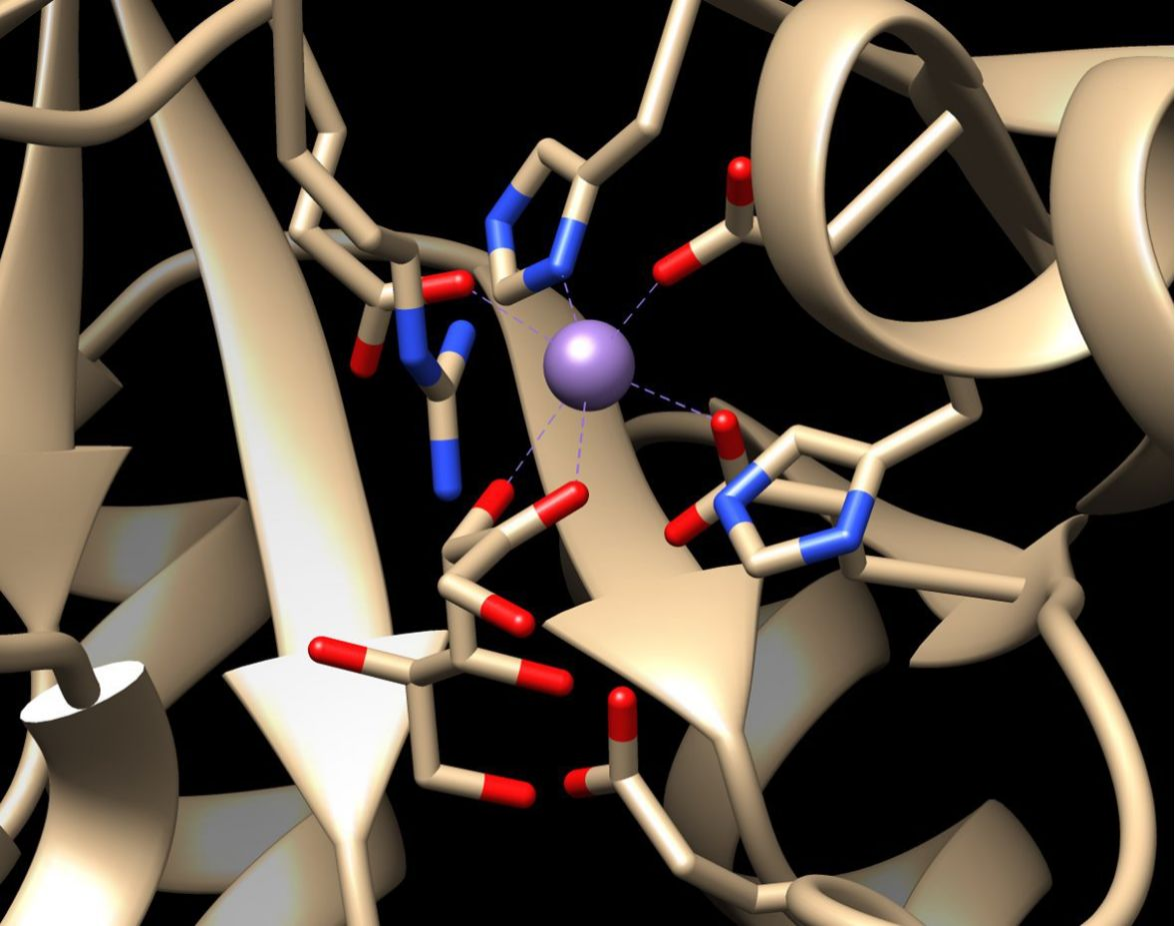
The active site in D-psicose 3-epimerase (DPEase) in the presence of D-fructose, showing the metal coordinating site and the substrate-binding site. The purple ball indicates the manganese(II) ion coordinating with key amino acid residues (Glu150, Asp183, His209, and Glu244) and the O-2 and O-3 of fructose. This figure was created using PDB File 2HK1 [38].

**Scheme 3:**
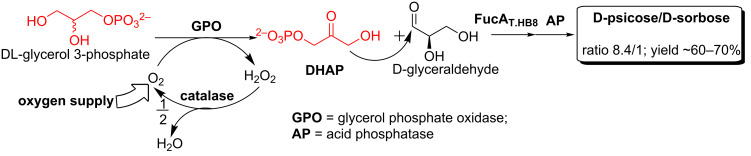
Enzymatic synthesis of D-psicose using aldolase FucA.

The enzymes described above are all generally named as DTEase family enzymes, despite the fact that all enzymes do not demonstrate strict specificity towards D-tagatose and low to moderate homology (20–60%) was found among them [37]. However, high similarities are observed for key amino acid residues in the active site, the metal coordinating site and the substrate-binding site of these enzymes (Figure 1) [37–38]. After all, the interconversion between D-fructose and D-psicose catalyzed by DTEase enzymes is an equilibrium process, thus large scale and high yield production of D-psicose as well as product purification remain problematic. Our lab mainly focuses on the in vitro production of rare sugars using dihydroxyacetone phosphate (DHAP)-dependent aldolases. In our previous work, we utilized L-fuculose-1-phosphate aldolase (EC 4.1.2.17) from *Thermus thermophilus* HB8 (FucA_T.HB8_) to stereoselectively synthesize D-psicose. The donor molecule DHAP, a highly expensive starting material ($970.00/250 mg, Sigma-Aldrich), was generated in situ from less expensive L-glycerol-3-phosphate or DL-glycerol-3-phosphate (Scheme 3) [39]. Such a one-pot multi-enzyme approach represents a cost-effective non-equilibrium pathway for the large-scale preparation of D-psicose (Scheme 3) [40].

#### D-Sorbose

**Scheme 4:**
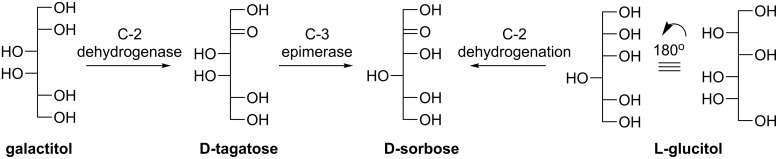
Proposed pathway of the D-sorbose synthesis from galactitol or L-glucitol.

D-Sorbose can be potentially utilized as a low-caloric sweetener [41], an insect control agent, and a starting material for producing industrially significant compounds [42]. D-Sorbose was reported to be prepared from galactitol catalyzed by *Pseudomonas sp.* ST 24 and the production yield was as high as 70%. The possible transformation route from galactitol to D-sorbose in this strain was deduced as follows (Scheme 4): the substrate galactitol is dehydrogenated at C-2 to afford D-tagatose followed by C-3 epimerization [43].

**Scheme 5:**
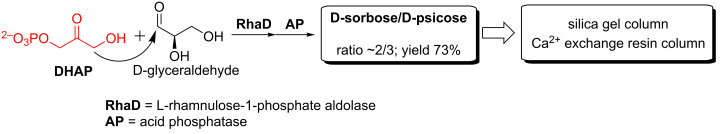
Simultaneous enzymatic synthesis of D-sorbose and D-psicose.

Izumori et al. established a method for the preparation of D-sorbose directly from D-tagatose with immobilized DTEase from *Pseudomonas sp*. ST-24 and 2 g of D-sorbose could be obtained from 3 g of D-tagatose. The conversion yield maintained at about 70% each time even if the batch was repeated five times [44]. Alternatively, D-sorbose could be prepared from L-glucitol via C-2 dehydrogenation through microbial conversion within a reasonable period of time (>95% yield and multigram scale, Scheme 4) [45–46]. The starting material L-glucitol could be obtained from the chemical reduction of a significantly cheaper starting material D-gulono-1,4-lactone instead of expensive L-glucose. In our previous work, we discovered that two rare sugars, D-sorbose and D-psicose, were simultaneously generated when L-rhamnulose-1-phosphate aldolase (RhaD, EC 4.1.2.19) [47] catalyzed the aldol addition between DHAP and D-glyceraldehyde (Scheme 5) [48]. Fortunately, the resulting diastereomers were easily separated with cation exchange resin (Ca^2+^ form) under elevated temperature to realize the preparative-scale production of both sugars [48–51].

#### L-Tagatose

**Scheme 6:**
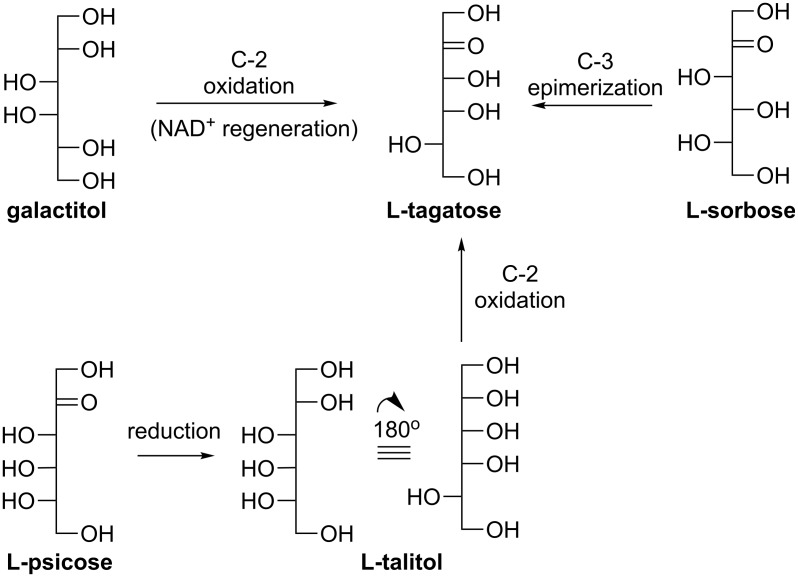
Biosynthesis of L-tagatose.

**Scheme 7:**
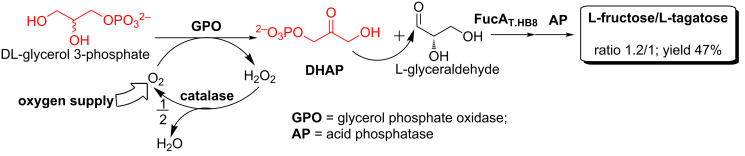
Preparative-scale synthesis of L-tagatose and L-fructose using aldolase.

Unlike its enantiomer D-tagatose which is a widely used rare sugar and produced in bulk quantity, L-tagatose has not yet been well studied or broadly utilized due to limited production pathway and high cost involved [52–53]. L-Tagatose can be synthesized via oxidation of galactitol (Scheme 6) by *Klebsiella pneumoniae* 40b (70% yield) [54]. Huwig et al. also reported an efficient oxidation of galactitol to L-tagatose through the use of galactitol dehydrogenase (GDH, EC 1.1.1.16) from *Rhodobacter sphaeroides* D and the overall yield was 78% [55]. This oxidation only requires catalytic amounts of NAD^+^, which is regenerated in situ through the reduction of pyruvate by L-lactate dehydrogenase (EC 1.1.1.27) [55]. Alternatively, Itoh et al. discovered that DTEase from *Pseudomonas sp.* ST-24 was a promising enzyme, which was active not only on D-ketohexoses but also on L-ketohexoses [56]. Therefore, L-tagatose was successfully prepared by immobilized DTEase using L-sorbose as the substrate although the production yield was only 20% (Scheme 6) [56]. Moreover, L-tagatose could be directly produced from L-psicose through microbial reduction followed by oxidation by *Enterobacter aerogenes* 230S (Scheme 6) and cells grown on xylitol have the best conversion potential [52]. The production yield could reach above 60% in the presence of glycerol [52] which may be responsible for NAD^+^/NADH regeneration. Large-scale preparation of L-tagatose has not been extensively studied yet due to elaborate routes or expensive starting materials. In our lab, the synthesis of L-tagatose was easily achieved by employing the previous discussed cost-effective one-pot four-enzyme system containing L-fuculose-1-phosphate aldolase (FucA, EC 4.1.2.17) (Scheme 7). In this case, L-glyceraldehyde was used as the aldol acceptor and L-tagatose and L-fructose were obtained simultaneously that can be easily separated and purified with cation exchange resin chromatography [39,57].

#### L-Fructose

**Scheme 8:**
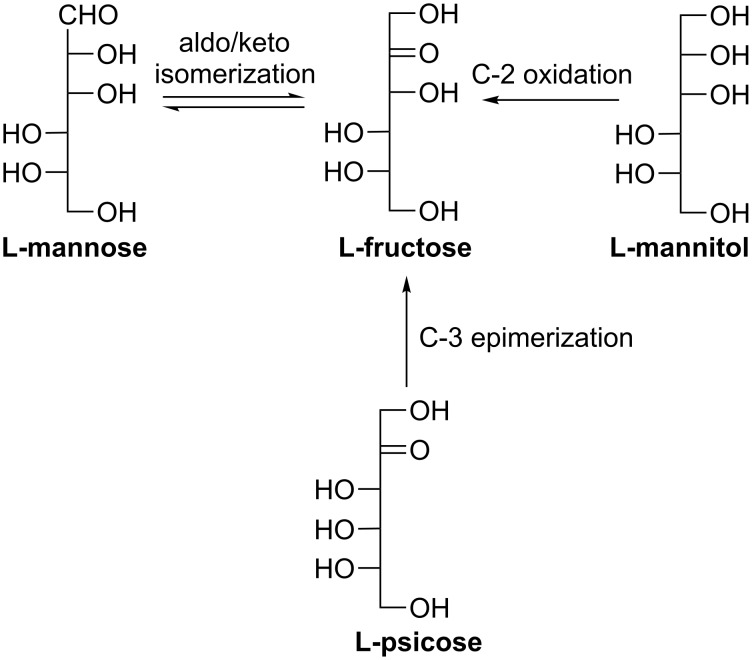
Biosynthesis of L-fructose.

**Scheme 9:**
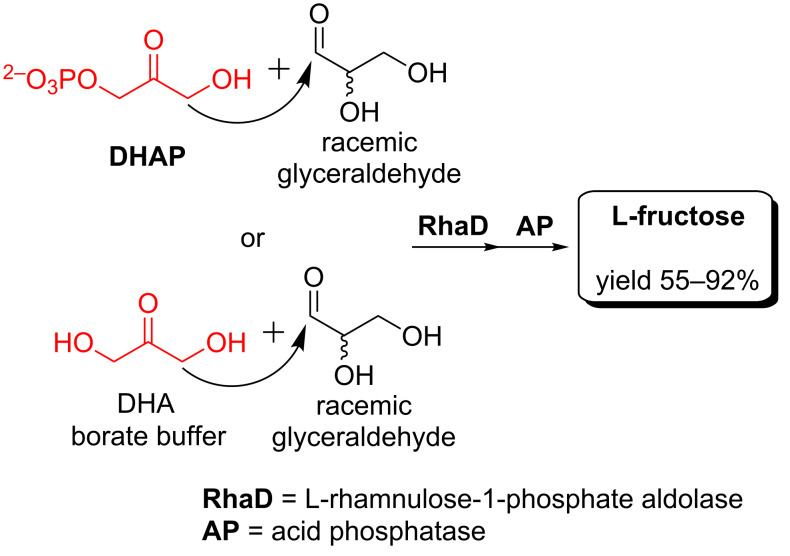
Preparative-scale synthesis of L-fructose using aldolase RhaD.

L-Fructose is a well known nonnutritive sweetener [9] and can be used as a potential inhibitor of several glycosidases [58]. Mayo et al. firstly established an enzymatic approach for the synthesis of L-fructose using L-mannose as a substrate through aldo–keto isomerization (Scheme 8) [59]. Dhawale et al. demonstrated another route via bacterial oxidation of L-mannitol (Scheme 8) [42]. Interestingly, the previously mentioned versatile DTEase from *Pseudomonas sp.* ST-24 could also catalyze the epimerization of L-psicose to produce L-fructose in a yield of 65% (Scheme 8) [56]. In addition, Franke et al. reported that the dihydroxyacetone phosphate (DHAP)-dependent aldolase L-rhamnulose-1-phosphate aldolase (RhaD) could selectively use L-glyceraldehyde out of racemic glyceraldehyde to produce L-fructose exclusively (55% yield) using expensive DHAP as the other substrate (Scheme 9) [60]. Sugiyama et al. further discovered that this aldolase RhaD could even tolerate dihydroxyacetone (DHA), instead of DHAP, as a donor substrate in the presence of borate buffer, which could function as a phosphate ester mimic in the aldolase-catalyzed reactions [61]. Based on this discovery, a practical one-step synthesis of L-fructose was established using DHA and racemic glyceraldehyde as substrates (Scheme 9) and the production yield was as high as 92% on a gram scale [61]. Our lab optimized their synthesis as well aiming to reduce the cost by employing the one-pot four-enzyme system as described before (also see Scheme 3 and Scheme 7) and obtained an overall isolated yield of 66% on a preparative scale [48,57].

#### 1-Deoxy-L-fructose

**Scheme 10:**
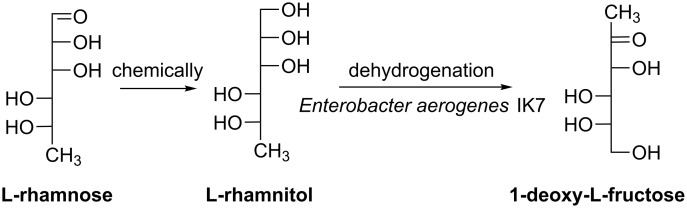
Chemoenzymatically synthesis of 1-deoxy-L-fructose [8].

Deoxy sugars play significant roles in central metabolism [62], cell signaling, immunological recognition and host–pathogen interactions [63–64]. Gullapalli et al. demonstrated an effective way of obtaining deoxy L-fructose by combining chemical and biotechnological approaches (Scheme 10) [8]. Hydrogenation of 6-deoxy-L-mannose (L-rhamnose) produced 6-deoxy-L-mannitol (L-rhamnitol) which was subsequently oxidized by *Enterobacter aerogenes* IK7 to afford the rare sugar 1-deoxy-L-fructose. The whole process was performed in an environmentally friendly fashion.

### II. Biosynthesis of rare aldohexoses

#### D-Allose

**Scheme 11:**
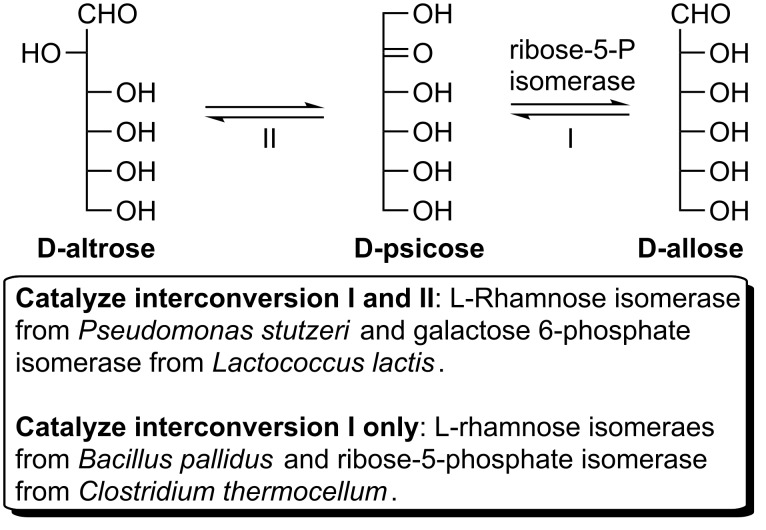
Potential enzymes (isomerases) for the bioconversion of D-psicose to D-allose.

D-Allose is the C-3 epimer of D-glucose or the aldo/keto-isomer of D-psicose (Scheme 11). Recently, increasing attention has been paid to this rare sugar due to its beneficial activities and potential pharmaceutical effects, such as antitumor [65], anticancer [66], anti-inflammatory [67], anti-oxidative [68], antihypertensive [69], cytoprotective [70], and immunosuppressant [71]. L-Rhamnose isomerase (EC 5.3.1.14), ribose-5-phosphate isomerase (EC 5.3.1.6), and galactose-6-phosphate isomerase (EC 5.3.1.26) were reported as potential enzymes for the bioconversion of D-psicose to D-allose [72]. L-Rhamnose isomerase from *Pseudomonas stutzeri* [73] and galactose 6-phosphate isomerase from *Lactococcus lactis* [74] catalyze two reversible isomerization reactions (interconversion between D-psicose and D-allose vs interconversion between D-psicose and D-altrose) with D-altrose as a byproduct. In contrast, L-rhamnose isomerase from *Bacillus pallidus* [75] and ribose-5-phosphate isomerase from *Clostridium thermocellum* [76] produce D-allose without D-altrose formation via only one type of isomerization. In addition, due to its thermostability and high conversion yield reported, ribose-5-phosphate isomerase from *Clostridium thermocellum* is the most suitable enzyme for the D-allose production.

**Scheme 12:**
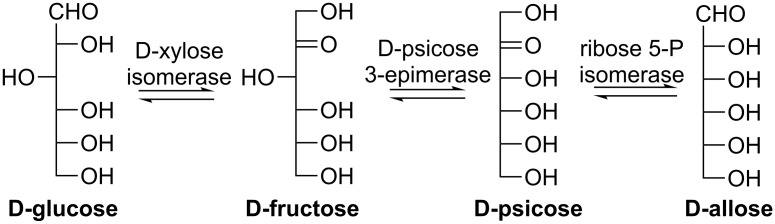
Three-step bioconversion of D-glucose to D-allose.

D-Allose can also be synthesized from inexpensive D-glucose as a starting material via a three-step bioconversion, sequentially catalyzed by D-xylose isomerase (EC 5.3.1.5), D-psicose 3-epimerase, and ribose-5-phosphate isomerase (Scheme 12). The overall conversion yield for the reaction sequence is expected to be higher compared to each step due to the promotion effect of the second and third step [72]. It remains a challenge to discover a D-allose epimerase which can directly epimerize D-glucose C-3 to afford D-allose because microorganisms do not generally consume such a rare monosaccharide for their growth.

#### L-Glucose

**Scheme 13:**
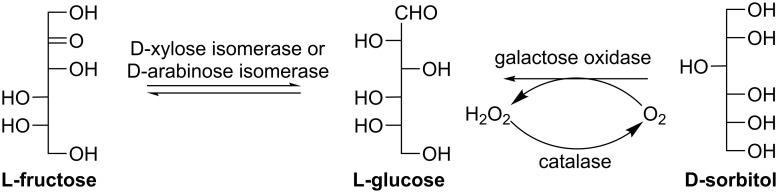
Biosynthesis of L-glucose.

L-Glucose does not occur naturally in higher living organisms, although it has many potential applications, such as a low calorie sweetener [77], a bulking agent [78], an inhibitor for bacterial growth [79] and various glucosidases. L-Glucose can also be used as an ideal starting material for glycoconjugate vaccines against the enteropathogenic bacterium *Shigella sonne* [80]. Enzymatically, L-glucose can be synthesized by isomerizing L-fructose (Scheme 13) catalyzed by D-xylose isomerase from *Candida utilis* [81] or whole cells of a mutant *Klebsiella pneumoniae* strain, which constitutively express D-arabinose isomerase (EC 5.3.1.3) [82]. An overall yield of 35% on a gram-scale was reported using this mutant strain [82]. Moreover, Yadav et al. immobilized galactose oxidase (EC 1.1.3.9) and catalase (EC 1.11.1.6) on a solid support (crab-shell particles or *Ocimum sanctum* seeds) and investigated their synthetic application for L-glucose production from D-sorbitol (Scheme 13) [83–84]. Catalase could degrade the hydrogen peroxide byproduct and increase the conversion by removing the inhibition effect of hydrogen peroxide toward galactose oxidase and regenerating oxygen.

#### L-Talose and D-gulose

**Scheme 14:**
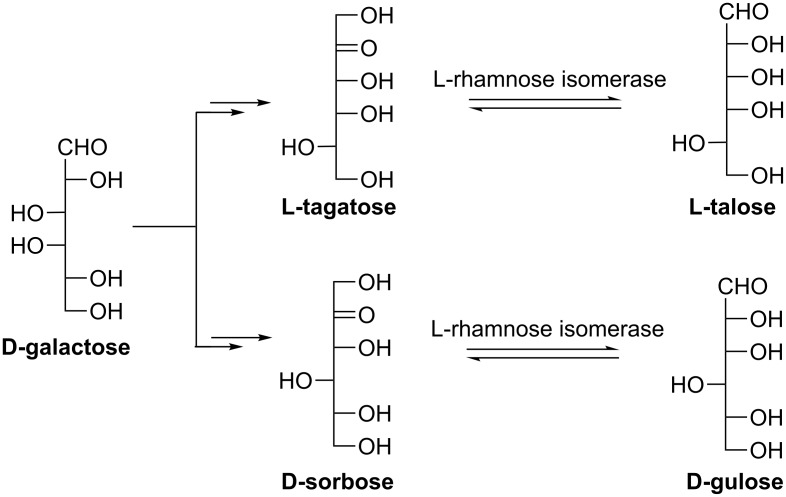
Enzymatic synthesis of L-talose and D-gulose.

The aldohexoses L-talose and D-gulose (C-3 epimer of D-galactose) are both very expensive owing to their scarcity in nature. L-Talofuranosyladenine, an adenine nucleoside derivative of L-talose, was found to be a slow-reacting substrate for calf intestinal adenosine deaminase and thus to inhibit the growth of leukaemia L1210 cells in vitro [85]. Crystalline D-gulose can be used as a drug-formulation agent and food additive. Bhuiyan et al. reported a simple enzymatic method for L-talose and D-gulose using immobilized L-rhamnose isomerase (EC 5.3.1.14) from *Pseudomonas sp.* strain LL172 (Scheme 14). Starting from rare sugars L-tagatose and D-sorbose (which were obtained from D-galactose via several steps of bioconversions), the production yields for L-talose and D-gulose were 12% and 10% respectively at equilibrium [86].

#### L-Galactose

**Scheme 15:**
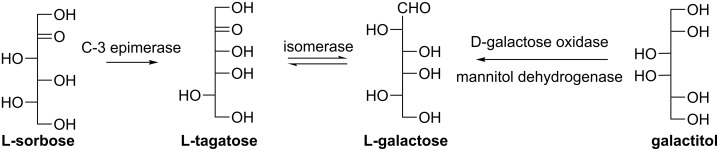
Enzymatic synthesis of L-galactose.

L-Galactose was reported to be an effective precursor of the L-ascorbate synthesis [87]. Leang et al. established an efficient two-step method for synthesizing L-galactose on a large scale from a common sugar L-sorbose, catalyzed by immobilized DTEase from a mutant *Pseudomonas sp.* ST-24 and recombinant L-rhamnose isomerase from *Escherichia coli* JM109 [88]. L-Sorbose was first epimerized by DTEase at C-3 to give L-tagatose (28% yield), which reached an equilibrium with L-galactose (~30%) catalyzed by the L-rhamnose isomerase (Scheme 15). L-Galactose was also directly synthesized by oxidizing galactitol using D-galactose oxidase [89], including the previously mentioned immobilized galactose oxidase [83–84], but the yields were generally low. In contrast, whole cells of recombinant *Escherichia coli* carrying a unique mannitol dehydrogenase (EC 1.1.1.255) represent a more scalable system [90] for oxidizing galactitol (Scheme 15). Alternatively, it was reported that L-galactose could be biosynthesized by epimerizing D-glucose through a novel but rare metabolic pathway during the biosynthesis of a sulfated L-galactan in the ascidian tunic, requiring a triple epimerization which brings about inversion of the configuration of carbon 2, 3, and 5 [91].

#### L-Fucose (6-deoxy-L-galactose)

**Scheme 16:**
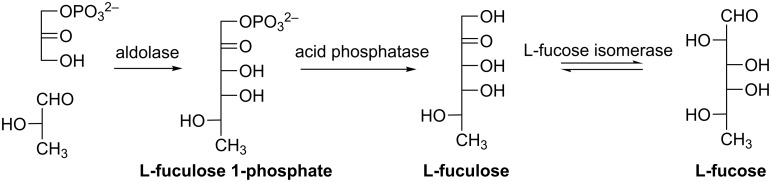
Enzymatic synthesis of L-fucose.

L-Fucose is a rare sugar belonging to the deoxy sugar family, which is a naturally occurring sugar widely found in biomass, especially in plant, but in minor amounts. It is also found on the mammalian cell surface and a fundamental core moiety of various carbohydrate antigens [92–93]. Microbiological synthesis of L-fucose is based on the fermentation by microorganisms, which generate L-fucose-containing exopolysaccharides (EPS). Then, L-fucose can be released by enzymatic hydrolysis and recovered from the hydrolysate [94]. Interestingly, metabolically engineered *Corynebacterium glutamicum* was used to produce guanosine-5’-diphosphate (GDP)-L-fucose, the precursor of fucosyl-oligosaccharides, from glucose and mannose [95]. L-Fucose (or its analogs) can also be prepared in vitro enzymatically in a three-step procedure (Scheme 16) [96]. Firstly, L-fuculose-1-phosphate (or the analogues) is synthesized by aldol addition catalyzed by L-fuculose-1-phosphate aldolase (FucA). Then, L-fuculose is produced after dephosphorylation by acid phosphatase (EC 3.1.3.2). Finally, L-fuculose is converted to L-fucose by L-fucose isomerase (EC 5.3.1.25). The procedure may be performed in a one-pot fashion or with purification after each step.

### III. Biosynthesis of rare sugar alcohols (hexitols)

Sugar alcohols are mainly derived from pentoses and hexoses. They are hydrogenated form of carbohydrates, in which the carbonyl group is reduced. Their flavor is like sucrose but they have less energy. Xylitol is very popularly used in the food industry while some other sugar alcohols such as allitol, D-talitol and L-sorbitol, are very expensive and unexploited. Here we summarize the biosynthesis of some hexitols derived from rare sugars.

#### Allitol

**Scheme 17:**
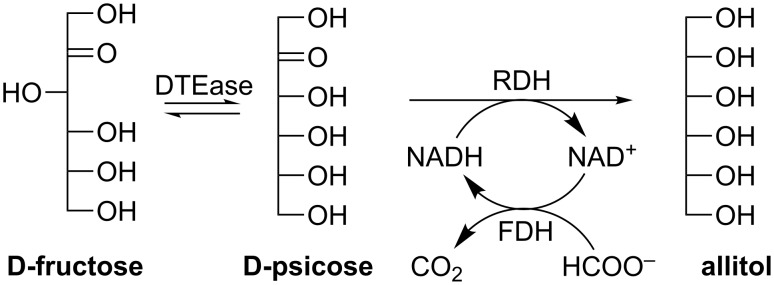
Synthesis of allitol from D-fructose using a multi-enzyme system.

Allitol can be prepared by reducing/hydrogenating the rare sugar D-psicose [97–98], which is derived from D-fructose via DTEase catalyzed conversion (Scheme 17). However, two problems exist in this pathway: low conversion rate for D-psicose and low D-psicose concentration available for bacterial reduction. These drawbacks could be overcome by a multi-enzyme system containing DTEase, ribitol dehydrogenase (RDH, EC 1.1.1.56) and formate dehydrogenase (FDH, EC 1.2.1.2) [99]. In this system, NADH is regenerated by an irreversible formate dehydrogenation reaction, promoting the conversion of D-psicose to allitol. As a result, the D-fructose and D-psicose equilibrium was shifted towards the formation of D-psicose and a complete conversion could be reached (Scheme 17).

#### D-Talitol

**Scheme 18:**
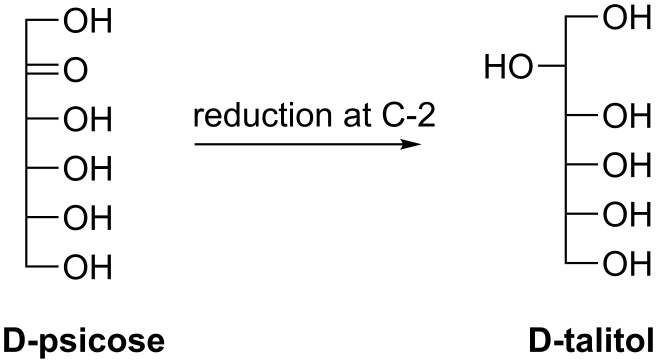
Biosynthesis of D-talitol via C-2 reduction of rare sugars.

D-Talitol is the other C-2 reduction product of D-psicose with a different stereospecificity to that of the allitol (Scheme 18). The conversion rate from D-psicose to D-talitol catalyzed by a halotolerant yeast strain *Candida famata* R28 could be significantly increased in the presence of various carbohydrates such as erythritol, D-sorbitol, ribitol and glycerol in the reaction mixture [100]. The faster consumption of the substrate could be explained by the in situ NADH regeneration while those supplementary carbohydrates are oxidized by a dehydrogenase. At 10% substrate concentration, the conversion rate could reach as high as 95% catalyzed by whole cells supplemented with 5% of D-sorbitol [100]. In addition, this bioconversion/reduction could also be carried out by various strains of *Mucoraceae* fungi [101]. However, D-talitol has not yet been well investigated for its applications due to the lack of a practical preparation method.

#### L-Sorbitol

**Scheme 19:**
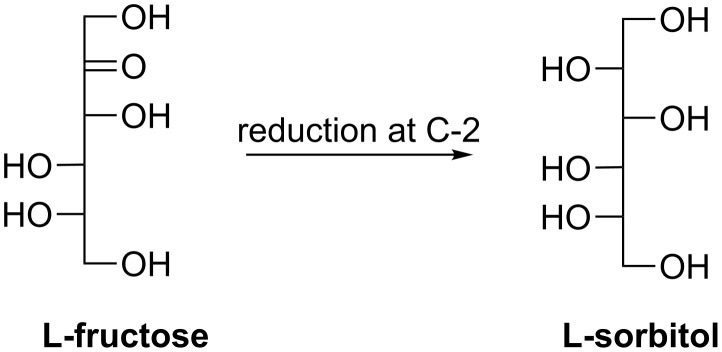
Biosynthesis of L-sorbitol via C-2 reduction of rare sugars.

L-Sorbitol was prepared from L-fructose (Scheme 19) catalyzed by whole cells of *Aureobasidium pullulans* strain LP23 and the conversion activity could be induced by L-arabinose [102]. Moreover, supplement of erythritol to the reaction mixture enhanced the conversion to L-sorbitol and one possible reason also lies in NADH regeneration resulting from erythritol dehydrogenation/oxidation.

## Conclusion

The preparation and functional study of rare sugars has become a hot topic because of their potential applications in different fields. An important factor restricting the utilization of rare sugars is their limited availabilities, resulting from limited synthetic methods. Biocatalysis often offers advantages over chemical synthesis, because enzyme-catalyzed reactions are often highly enantioselective and regioselective. In addition, enzymatic reactions are usually performed under mild conditions and are environmentally friendly. However, two major challenges remain in this endeavor: 1) microorganisms and related enzymes presently applied in rare sugar synthesis are from non-generally regarded as safe (GRAS) sources, which may stimulate an increasing demand for introducing related enzymes into GRAS microorganisms; 2) many biosynthetic methods involve a chemical equilibrium (isomerization or epimerization) between two or more sugars that may lead to low to moderate yields and difficulties in product purification. Advancements in genomics and bioengineering can potentially solve this problem by improving the properties (activity, thermostability, or substrate-binding affinity) of existing enzymes or discovering new systems and robust biocatalysts for industrial-scale applications.
